# Unlawful killing at inquests: Clarity or confusion?

**DOI:** 10.1177/00258172221099080

**Published:** 2022-06-13

**Authors:** Gerard Kelly

**Affiliations:** Lecturer in Law, Queen’s University Belfast, UK

**Keywords:** *Maughan*, unlawful killing, standard of proof, inquests, coroners law, coronial jurisdiction

## Abstract

This article examines the standard of proof for unlawful killing in coronial proceedings. Historically, the criminal standard of proof governed inquest findings of unlawful killing. In *R (Maughan)* v *Her Majesty’s Senior Coroner for Oxfordshire*, the Supreme Court resolved the important question of whether the criminal or civil standard governed inquest conclusions of unlawful killing. The court concluded that the correct standard of proof for all conclusions in coronial proceedings is the balance of probabilities. This article argues that whilst preserving differing standards of proof in coronial proceedings was no longer defensible and *Maughan* has provided welcome clarity, unanswered questions remain concerning the implementation of this fundamental change.

## Introduction

The standard of proof governing inquest verdicts of unlawful killing has been heavily influenced by the historical nature of inquests. In the past, English coronial proceedings were also means for finding criminal liability, but today are concerned only with the investigation of deaths. However, despite this significant shift in the role and scope of coronial proceedings, until recently there has been little judicial consideration of how this impacted the quantum of proof required to conclude that a deceased was killed unlawfully. In *R (Maughan)* v *Her Majesty’s Senior Coroner for Oxfordshire*, the UK Supreme Court considered this question and determined that the correct standard of proof for all conclusions in coronial proceedings is the balance of probabilities.

## Background

On 11 July 2016, the appellant’s brother, James Maughan, a prisoner at HMP Bullingdon, was found hanging in his cell. At the inquest the coroner decided, with reference to the criminal standard of beyond reasonable doubt, that the evidence was insufficient to allow the jury to safely reach a short form conclusion of suicide. The coroner instead posed questions to the jury and invited the jury to enter a narrative statement of the circumstances of the deceased’s death on a balance of probabilities. The jury answered the questions by saying that the deceased had a history of mental health issues and, on the balance of probabilities, that he had intended to fatally hang himself and that additional vigilance would not have prevented his death.

The coroner’s direction to the jury was informed by the statutory framework: in particular, the Coroners (Inquests) Rules 2013 (“2013 Rules”) and the record of inquest, a prescribed form which appears in the Schedule to the 2013 Rules. Note (iii) to the record of inquest provides that:

“The standard of proof required for the short form conclusions of ‘unlawful killing’ and ‘suicide’ is the criminal standard of proof. For all other short-form conclusions and a narrative statement the standard of proof is the civil standard of proof.”

Mr Maughan’s brother subsequently issued judicial review proceedings challenging the record of inquest, alleging that the coroner had misdirected the jury on the appropriate standard of proof.

The High Court concluded that the correct standard of proof concerning suicide determination was the civil standard and held that this applied in respect of both short form and narrative conclusions.^1^ This effectively ended what had been described as the hybrid approach, as reflected in Note (iii). Mr Maughan’s brother appealed.

The Court of Appeal upheld the High Court’s decision.^2^ First, the court found no justification in having a “hybrid” approach and held that the same standard must apply in both short form and narrative conclusions. Second, the court considered what that standard should be. Davis LJ discussed the historical rationale for applying the criminal standard for suicide determinations, acknowledging the historical criminalisation of suicide, before noting that, whilst findings of suicide could be “dreadfully upsetting” for the bereaved family, it is no longer a crime.^3^

Davis LJ further elaborated that there was no discernible reason why a criminal standard of proof should apply drawing an analogy with the civil courts which “generally apply in civil proceedings the civil standard”.^4^ Mr. Maughan subsequently appealed to the UK Supreme Court. Although the case directly concerned suicide, Davis LJ observed that it would be “wrong” not to comment “on the standard of proof applicable to cases at inquests where the issue of unlawful killing arises”.^5^ Whilst acknowledging that there was a “very powerful case for saying that the civil standard of proof should apply to all inquests in all respects”,^6^ Davis LJ concluded that the current state of the law was that the criminal standard governed inquest conclusions of unlawful killing.^7^

The central issues for the Supreme Court involved determination of the correct standard of proof for a coroner’s inquest to conclude that a deceased had died by suicide and whether the same standard should apply to both short form and narrative conclusions.^8^ By a 3–2 majority, the Supreme Court held that the civil standard was the correct standard and that it should apply to both short form and narrative conclusions. Writing for the majority, much of Lady Arden’s reasoning focused on the correct interpretation of Note (iii) in the record of inquest form, as set out in the Schedule to the 2013 Rules.^9^ She concluded that a note to a form in a statutory instrument would not be the place for a substantive change to the law, such as the standard of proof.^10^

After resolving that the governing standard of proof for suicide determination was the civil standard, Lady Arden turned to consider whether the criminal standard should be retained for unlawful killing. She observed that “the short form conclusions of unlawful killing and suicide cannot satisfactorily be distinguished with respect to the standard of proof”,^11^ before concluding that “a common standard applying to both unlawful killing and suicide is more consistent with principle and removes an inherent inconsistency in the determinations made at an inquest”.^12^

## Commentary

Prior to *Maughan*, English law required the criminal standard of proof for short form conclusions of suicide and unlawful killing. Post-*Maughan* the standard of proof for all conclusions at inquests is now the civil standard. As a consequence of lowering the standard of proof, it seems likely that there will be an appreciable increase in unlawful killing conclusions at inquests. Yet, in the aftermath of *Maughan*, the Chief Coroner considered the potential implications for unlawful killing inquest conclusions and stated that:

*“*In 2019 there were fewer than 166 conclusions of unlawful killing made by coroners or juries in inquests out of a total number of 31,284 inquest conclusions, or approximately 0.5%. Although the decision in *Maughan* will probably have some continuing impact on the figures, the issue of unlawful killing is likely to feature in relatively few cases.”^13^

It is doubtful whether the Chief Coroner’s forecast that unlawful killing will feature in “relatively few cases” will prove accurate. Instead, it seems reasonable to predict a material upward trend in unlawful killing conclusions.

The criminal standard had historically applied due to the very serious consequences of an unlawful killing verdict. In the past, coronial proceedings could determine questions of criminal liability and so, in such instances, the higher quantum of proof of beyond reasonable doubt was both a sensible and necessary safeguard. However, as Lady Arden emphasised, “[i]nquests are concerned today not with criminal justice but with the investigation of deaths”.^14^ That said, as Lady Arden also acknowledged, a person implicated in a finding of unlawful killing will suffer prejudice, but she observed that in such instances an individual would have been equally liable to suffer prejudice from findings by way of a narrative statement (which were, pre-*Maughan*, already governed by the lower civil standard).

Lady Arden’s over-arching focus was to advance a system of fact-finding which was both internally consistent and principled. There can be little doubt that – judged by this metric – *Maughan* delivers a more coherent coronial framework by promoting consistency of approach within coronial proceedings and removing the internal inconsistency produced by having different rules for short form and narrative conclusions. In these respects, the clarity which *Maughan* provides is very welcome.

However, *Maughan* is also proving the source of some confusion too. Lady Arden’s reasoning heavily emphasised the inquisitorial and civil nature of coronial proceedings. Her classification of coronial proceedings as a form of civil proceedings was contested by the minority. In his dissent, Lord Kerr, with whom Lord Reed agreed, considered coronial proceedings as neither civil nor criminal, but rather “sui generis proceedings with rules of procedure of their own”.^15^ Indeed, in this respect, Lord Kerr’s perspective was also consistent with that of Davis LJ in the Court of Appeal, who had observed that:

*“*It is elementary, but nevertheless essential to emphasise in view of the issues arising on this appeal, that inquests are not to be regarded as litigation. They are not. They are not criminal proceedings. They are not civil proceedings.”^16^

Paradoxically, by introducing a lower standard of proof for determinations of unlawful killing, *Maughan* may well accentuate the adversarial element of coronial proceedings which Lady Arden had sought to downplay. A finding of unlawful killing is likely to remain, as Lord Kerr put it, a “solemn pronouncement” with resonance far beyond a narrative conclusion on the facts. Indeed, the Chief Coroner has reiterated the “intrinsic gravity” of such a finding in his most recent guidance concerning unlawful killing.^17^ Therefore, it is not unreasonable to expect that some interested persons – such as corporate employers of an employee who died at work – will be particularly attuned to the risk of adverse reputational implications. It is long-established law that an inquest conclusion has no legal bearing on criminal proceedings,^18^ but corporate interested parties may well consider that this provides insufficient protection. There may also be increased recourse to judicial review challenging coronial decisions.

Relatedly, *Maughan* may have unanticipated public policy consequences including questions concerning funding (or the lack thereof) and the impacts on bereaved families. It is notable, for example, that the Ministry of Justice’s 2019 report, “Review of Legal Aid for Inquests”, stated that:

“When people raise concerns about inquests becoming more adversarial they mean that the approach adopted by lawyers representing … ‘interested persons’ is more like that of the prosecution and defence in a criminal trial, which might be characterised as point-scoring – rather than assisting the coroner to get to the truth – and that this is having an adverse impact on bereaved families.”^19^

Section 10 of the Coroners Act 2009 prohibits inquests from reaching conclusions concerning criminal liability on the part of a named person. In addition, Schedule I of the 2009 Act sets out circumstances when a coroner must suspend and resume inquest investigations. For example, under paragraphs 1 and 2 of Schedule I, a coroner must suspend an investigation and adjourn an inquest if a person has been or may be charged either with a homicide offence involving the death of the deceased or an offence alleged to be a related offence. The coroner also has a much broader discretionary statutory power under paragraph 5 of Schedule I to suspend an investigation if s/he considers it appropriate, where other criminal or civil proceedings arise from the same facts. Lady Arden considered the operation of these provisions as an important protection “against a short form conclusion reached on a civil standard which is unfavourable”.

Paragraph 8(5) of Schedule I provides that a determination under the resumed inquest may not be inconsistent with the outcome of the proceedings in respect of the charge by reason of which the inquest was suspended. This would appear to suggest that a resumed inquest could not reach a conclusion of unlawful killing if a jury has already found the individual not guilty in a criminal trial for homicide offences. However, the Chief Coroner has instead interpreted this to provide that “[i]f in such an inquest the coroner or inquest jury find that the requisite elements of murder, manslaughter or infanticide are established on the balance of probabilities then a conclusion of unlawful killing will be permissible even though there has already been an acquittal of the offence following a homicide trial”. This is likely to further emphasise the adversarial dimension of inquests, rather than the inquisitorial qualities which Lady Arden had recognised and which had influenced her reasoning in classifying coronial proceedings as a form of civil proceeding.

## Concluding Thoughts

The historical context provides an explanation as to why the criminal standard of proof to conclude that a deceased was unlawfully killed was once a prudent protection. For inquests today, by standardising the quantum of proof, *Maughan* has provided welcome clarity to the process governing coronial findings. Inquests should now be less likely to produce anomalies, and findings – both short-form and narrative – will be more consistent with principle. Yet there are unresolved and potentially unanticipated (or, at least, under-appreciated) considerations too. In the outworking of *Maughan*, the Chief Coroner has confirmed that coronial findings may diverge from verdicts in a criminal trial. This interpretation of the statutory safeguards contained in Schedule I is unlikely to reassure corporate interested parties in circumstances where, for example, an employee died at work. Moreover, whilst an inquest is an inquisitorial process and not a trial, as a consequence of *Maughan* there may be heightened focus on the adversarial features of the coronial process and, relatedly, increasing pressure to implement more broadly accessible public funding arrangements.

## Notes

1 *R (Maughan)* v *Her Majesty’s Senior Coroner for Oxfordshire* [2018] EWHC 1955 (Admin), 1 All ER 561.

2 *R (Maughan)* v *Her Majesty’s Senior Coroner for Oxfordshire* [2019] EWCA Civ 809, [2019] QB 1218.

3 Ibid, [74].

4 Ibid.

5 Ibid, [90].

6 Ibid, [91].

7 Ibid, [94].

8 *R (Maughan)* v *Her Majesty’s Senior Coroner for Oxfordshire* [2020] UKSC 46, [2021] AC 454.

9 Ibid, [19]–[57].

10 Ibid.

11 Ibid, [93].

12 Ibid, [96].

13 The Coroners’ Society of England & Wales, “Chief Coroner: Law Sheet No 6 – On the Application of *R (on the application of Maughan)* v *HM Senior Coroner for Oxfordshire* [2020] UKSC 46” (2021) <https://www.coronersociety.org.uk/_img/pics/pdf_1610640613-843.pdf> (last checked 1 February 2022).

14 *R (Maughan)* v *Her Majesty’s Senior Coroner for Oxfordshire* [2020] UKSC 46, [2021] AC 454 [81].

15 Ibid, [141].

16 *R (Maughan)* v *Her Majesty’s Senior Coroner for Oxfordshire* [2019] EWCA Civ 809, [2019] QB 1218 [25].

17 Chief Coroner, Law Sheet No 1, “Unlawful Killing”.

18 The conclusion of a coroner is strictly inadmissible as to the truth of its contents in later proceedings (*Hollington* v *F Hawthorn & Co Ltd* [1943] KB 587).

19 Ministry of Justice. Final Report: Review of Legal Aid for Inquests (2019) https://assets.publishing.service.gov.uk/government/uploads/system/uploads/attachment_data/file/777034/review-of-legal-aid-for-inquests.pdf (last checked 1 February 2022)

